# SAO2Vec: Development of an algorithm for embedding the subject–action–object (SAO) structure using Doc2Vec

**DOI:** 10.1371/journal.pone.0227930

**Published:** 2020-02-05

**Authors:** Sunhye Kim, Inchae Park, Byungun Yoon

**Affiliations:** 1 Department of Industrial & Systems Engineering, College of Engineering, Dongguk University, Seoul, SOUTH KOREA; 2 College of IT Engineering, Hansung University, Seoul, SOUTH KOREA; University of Sao Paulo, BRAZIL

## Abstract

In natural-language processing, the subject–action–object (SAO) structure is used to convert unstructured textual data into structured textual data comprising subjects, actions, and objects. This structure is suitable for analyzing the key elements of technology, as well as the relationships between these elements. However, analysis using the existing SAO structure requires a substantial number of manual processes because this structure does not represent the context of the sentences. Thus, we introduce the concept of SAO2Vec, in which SAO is used to embed the vectors of sentences and documents, for use in text mining in the analysis of technical documents. First, the technical documents of interest are collected, and SAO structures are extracted from them. Then, sentence vectors are extracted through the Doc2Vec algorithm and are updated using word vectors in the SAO structure. Finally, SAO vectors are drawn using an updated sentence vector with the same SAO structure. In addition, document vectors are derived from the document’s SAO vectors. The results of an experiment in the Internet of things field indicate that the SAO2Vec method produces 3.1% better accuracy than the Doc2Vec method and 115.0% better accuracy than SAO frequency alone. This proves that the proposed SAO2Vec algorithm can be used to improve grouping and similarity analysis by including both the meanings and the contexts of technical elements.

## 1. Introduction

Given the sophistication of the information society and a large amount of technical literature being created, it is quite important to analyze the implications of that literature. In general, technical documentation is written to record scientific or technical knowledge; this includes patent literature, technical reports, and product descriptions. These technical documents contain ample information regarding science and technology, as well as practical examples and trends; this information can be processed and used for various purposes.

Many text-mining researchers have suggested approaches for extracting important content from documents. At the beginning of this field, researchers used data such as the number of publications, number of patent quotes, and bibliographic data to analyze technical documents [1]. This analysis is based on the publication dates and publishers of technical articles such as patents and papers. Such bibliographic data can be used in forecasts and have the advantage of the clarity of information [2], but they are limited in that they do not include the content of the articles. Therefore, to also consider the content, text-mining techniques can be applied to derive critical information from the technical documents’ text. These techniques are some of the most popular methods of data analysis, as they can be used to extract useful information from unstructured textual data and can effectively express that information so as to derive analytical results from the textual context on a macro scale [3–6]. Typical text-mining applications include searching, information retrieval, document clustering, document classification, information extraction, and document summarization. Many studies have been conducted to solve these text-mining application tasks. In particular, the framework based on the relationship between concept frameworks has been proposed from the same point of view with this study. [7–11]. For these text-mining applications, natural language processing (NLP) must be performed [12]. In one NLP technique, the subject–action–object (SAO) structure is used to extract information objective, structure, and effect data from the unstructured text before converting that information into structured textual data [13]. The SAO structure is of particular use in the analysis of technical documents. In the past, researchers often used keyword-based analysis—a method based on frequently appearing words—to conduct content analyses [14,15]. However, such keyword-based analysis is limited in that it tends to overlook the meanings of verbs and in that it emphasizes qualitative, expert-based information [14,16]. Therefore, analysis based on the co-occurrence of words is now widely used [17–19]; this method has allowed analyzers to understand texts and subjects more quickly than they could use any of the previous analysis methods [20]. However, this analysis method is limited in that it can not identify specific relationships between words and in that it can not properly judge whether high-frequency words or general terms are relevant to the topic. Analysis based on co-occurrence also has a limitation because it is not specific to technical documents.

Although SAO structures are suitable for the analysis of technical documents (because they can be used to extract the key details of a technology), they do not account for how the omission of certain words can indicate various contextual relationships within sentences (or documents). Therefore, activities that use extracted SAO structures, such as network analysis and technology-tree analysis, require many manual processes to ensure a correct understanding of the relationships between SAO structures. In addition, analyses that use only the frequency of SAO appearance also require manual definitions of the steps. Furthermore, analyses that use existing SAO structures are limited to analyses of large amounts of data because the structures are not quantified. Therefore, we propose using an SAO embedding algorithm to shorten this qualitative process and to allow for richer and quicker analyses by determining the relationships among SAOs and by including the context of the omitted SAO structures. The basic idea of embedding SAO involves using the Doc2Vec algorithm.

Doc2Vec is a word- and sentence-embedding NLP algorithm that is based on distributed and symbolic data representations called neural-net language models [21–23]. Doc2Vec has the advantage of allowing for the quick analysis of large amounts of data by expressing words in the vector-space model and by considering the context (based on the co-occurrence of words) when learning [21]. However, since Doc2Vec places the same weights on all the words in a document, its effectiveness is limited to finding a general word vector; it does not express particular topics well [24,25]. Therefore, although this paper used co-occurrence (i.e., the basic idea of Doc2Vec) and Doc2Vec’s objective function to update the weight matrix, we also added an updating process based on the SAO structure. The proposed approach can be used to quickly obtain the vectors of SAOs and documents. The quantified SAO vector implies the existence of a relationship between SAOs, and this can complement the limitations of the existing methods. In addition, since both the SAO vectors and document vectors have technical functions, they may be helpful in overcoming the limitations of the Doc2Vec vector. In summary, the result of SAO2Vec is expected to reflect the meaning of a technology’s function and context within a document, thus enabling an analysis of the relationship between technological elements and SAOs for large volumes of data.

In this paper, we consider Internet of things (IoT) technology as a means to validate the proposed approach. In this technology, all everyday objects are connected to the Internet. According to BMI Research, by 2050, at least 40 billion objects from all parts of life will be connected to the Internet. Most companies (57%) have already introduced IoT technology, and by 2019, this rate is expected to reach 85%, with the IoT market expected to grow at an annual rate of 21.8% through 2022 [26]. Core technologies in the IoT field include sensing, wireless communications, networking, and service interfaces [27]. As shown in Table 1, we selected wireless communications and network technologies as the subject area for this analysis because these areas have high rates of growth and patent registration.

**Table 1:** List of patents registered by CPC, 2017 [28].

Rank	CPC code	Register Num (2017)	Register Num (2016)	Growth Rate
1	G06F^a^	48935	47919	2.12%
2	H04L^b^	33575	31414	6.88%
3	H01L^c^	26989	26741	0.93%
4	H04W^d^	21258	19390	9.63%
Average Growth Rate				2%

^a^CPC code “G06F” is about electrical digital data processing.

^b^CPC code “H04L” is about transmission of digital information.

^c^CPC code “H01L” is about semiconductor devices; electric solid state devices.

^d^CPC code “H04W” is about wireless communications networks.

The rest of this study is organized as follows. Section 2 is a summary of the related studies, and Section 3 includes the framework for using the Doc2Vec algorithm and SAO structure. In Section 4, the proposed algorithm is applied to the patents in the IoT field. Section 5 provides discussions of this method and its implications. Finally, the conclusions and suggestions for future works are presented in Section 6.

## 2. Backgrounds

### 2.1 SAO structure

As mentioned in the Introduction (Section I), scholars have widely used the SAO structure to analyze technical documents such as patents. This structure uses verbs to explain the functions of technology; this has the advantage of being able to clearly represent the relationships between technical elements, and it can be used in fields such as technological analysis and patent-similarity analysis [29,30]. In this context, researchers have attempted to use the SAO structure to meet needs in a variety of areas, including patent-network analysis [31], trend analysis [30,32], merger and acquisitions (M&A) target selection [33], technology-tree analysis [34], and technological-component identification [35].

The SAO-structure technique involves extracting the objective, structure, and effect information from unstructured textual data and then converting that information into structured textual data. This technique is helpful in the analysis of technical documents such as patents [13]. A scientist from the former Soviet Union, Genrich Altshuller, first proposed its use in function analysis based on theory of solving inventive problem (TRIZ), a collection of 40 tools (including function analysis and division) that allow for vast amounts of engineering and scientific knowledge to be applied to engineering problems [36]. In function analysis, the entire system is structured in terms of subjects, actions, and objects in order to idealize the problem of the system’s functions. The SAO structure illustrates the relationship between means and purposes. It can also be used to identify specific relationships between words and topics or to determine which words best express certain concepts [13,29]. If the action and object describe a technical problem and the subject indicates a solution, then the relationship between the problem and the solution can be inferred [37]. Therefore, the SAO structure can be used to determine the key findings regarding a technology, can properly represent the relationship between elements of that technology, and can present a variety of technical information [34,38]. For this reason, many researchers have begun to use SAO structures in technical analyses. Generally, they have used the frequency of SAOs or the similarity among SAOs. These scholars have studied the frequency of SAOs by extracting SAO structures from patents or technical documents and then calculating the frequency with which the same (or similar) SAOs appear. These researchers then use this information to build technology trees or maps and to conduct trend analyses [30,31]. To determine the similarity of patent documents using SAO structures, researchers have extracted the SAO structures and performed semantic-equivalence calculations based on dictionaries (or similar calculations using fuzzy matching). In addition, using calculated similarity, researchers have also conducted technical-performance, technological-roadmap, and technological-trend analyses so as to identify the key elements of given technologies [32–35]. However, these scholars had to manually define each relationship between SAOs and had to spend a lot of time processing large amounts of unquantified data. Although SAO includes sentences’ key technical elements, it is also limited in that it treats different sentences with the same SAO structure as the same; SAO alone does not consider the context or modifiers. Therefore, in this study, our aims are to use SAO structures to develop text-mining techniques that can obtain rich information about technical functions and context, as well as to quantify and automate these techniques to facilitate the analysis. For this purpose, the SAO structures are extracted from technical documents using an extraction algorithm that is based on the simplest of the various algorithms for extracting SAO structures.

### 2.2 Word embedding and document embedding

In text mining, word embedding and document embedding are the most frequently used NLP techniques for mapping a word or document vector in vector space [39,40]. The resulting vector of words, sentences, and documents quantifies the (typically unstructured) textual data so that quantitative analysis methods can be used. This enables the application of traditional quantitative data analysis methodologies (e.g., regression and correlation) to textual data and also reduces the number of expert manual tasks, thereby speeding up the processing of large amounts of data [41]. Mikolov suggested that word vectors and sentence vectors could achieve better NLP performance when similar words or sentences are grouped and noted that the computation of word vectors and sentence vectors enables richer analysis [21,22].

Researchers have identified many methods for embedding words, including the initial one-hot vector methods. An one-hot vector creates a N-dimensional vector to express a particular word in a dictionary of N words. This method is only concerned with the occurrence of a word, so there is a substantial loss of information. Scholars have since proposed several alternative methodologies, such as count-based and predictive methodologies. Count-based methodologies include count vectors that use only a word’s frequency, as well as term frequency inverse document frequency (TF-IDF) vectors that can use the information regarding how often given words occur in specific documents. A version of Word2Vec is used in the predictive methodology. Word2Vec has two models: the continuous bag of words (CBOW) model, which uses context to predict current words, and the skip-gram model, which predicts peripheral words using current words. Scholars at first used document embedding as a one-hot vector methodology similar to word embedding. They gradually elaborated upon this approach to create a synthetic operator that synthesizes a word vector (which is obtained through embedding a word into a document vector) [21, 42–44]. This method has limitations in that its accuracy varies greatly depending on the accuracy of the results of the word embedding, as the accuracy of the word vector can not be considered. Thus, the Doc2Vec (or Paragraph2Vec) algorithm, which expands upon the basic ideas of the Word2Vec algorithm, has since come into wide use.

**Fig 1:**
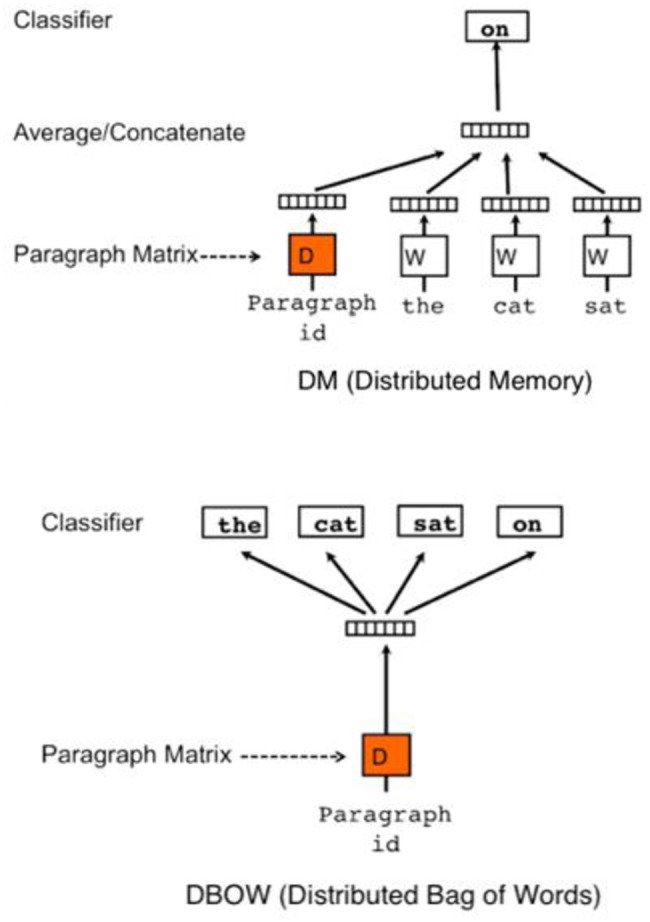
Doc2vec models: DM model, Dbow model [14].

Fig 1 shows Doc2Vec’s two models: the distributed-memory model and the distributed-bag-of-words model. Using the Doc2Vec algorithm (which acts as a memory cell that remembers information that is not reflected in the context of Word2Vec [21]), various vectors can be derived for the word vector, the sentence, and the document. This helps to compensate for the traditional method’s limitations (such as its inability to consider the words’ sequence) and allows for words, sentences, paragraphs, and documents to be mapped in the same space. Researchers have used this methodology in a lot of studies because it can be applied to word sequences of various lengths and can result in a document vector that considers the context. A variety of text embedding methodologies such as Glove, FastText, ELMO, and BERT were also proposed after Word2Vec was suggested. Glove’s training objective is to learn the data in order to make the scalar product of two embedded vectors equal the logarithm of the words’ probability of co-occurrence in overall corpus [45]. It makes easier to measure the similarity between embedded word vectors, and better to reflect statistical information across the corpus. FastText is a methodology for considering words as Bag-of-Characters and embedding n-gram characters rather than individual words [46]. Therefore, each word is represented by the sum of the embedded n-gram. ELMO operates in the form of performing word embedding through a traditionally trained Bilstm neural network, and the same word can be embedded as a different vector depending on the context [47]. BERT, the latest model, is a language representation model that is based on a transformer that transfers the learning process into the labeled data analysis with specific tasks, after pre-training the model with large amounts of unlabeled data [48]. However, although these methods are effective at obtaining general word vectors, they do not properly reflect the technological characteristics of a technical document, because these models do not consider which words are technically important in a sentence. Therefore, we propose using SAO2Vec—which is based on the idea behind Doc2Vec (co-occurrence). This produces a vector that acts as a memory cell for words. Eq 1 is the objective function of Word2Vec and Doc2Vec; this function produces the probability that *w*_*c*,*j*_ is actually observed in the output of the word *w*_*i*_ when a given input is entered. In this paper, we determined the meaning and the function of the SAO structure using this objective function.

$$p(w_{c,j}=w_{O,c}|w_{I})=y_{c,j}=\frac{exp\left(u_{c,j}\right)}{\sum_{j^{'}=1}^{V}exp\left(u_{j^{'}}\right)}$$ (1)

## 3. Research framework

The proposed SAO2Vec algorithm is a text-mining technique that can be used to textually process technical documents. Using SAO2Vec, it is possible to obtain document and SAO vectors (based on Doc2Vec and the SAO structure). The Doc2Vec algorithm can consider the meaning of context, and the SAO structure allows for a better understanding of the meaning of the expressed functions. Vectors obtained using SAO2Vec, therefore, can properly reflect the technical elements and context of the document.

### 3.1 Overall process

**Fig 2:**
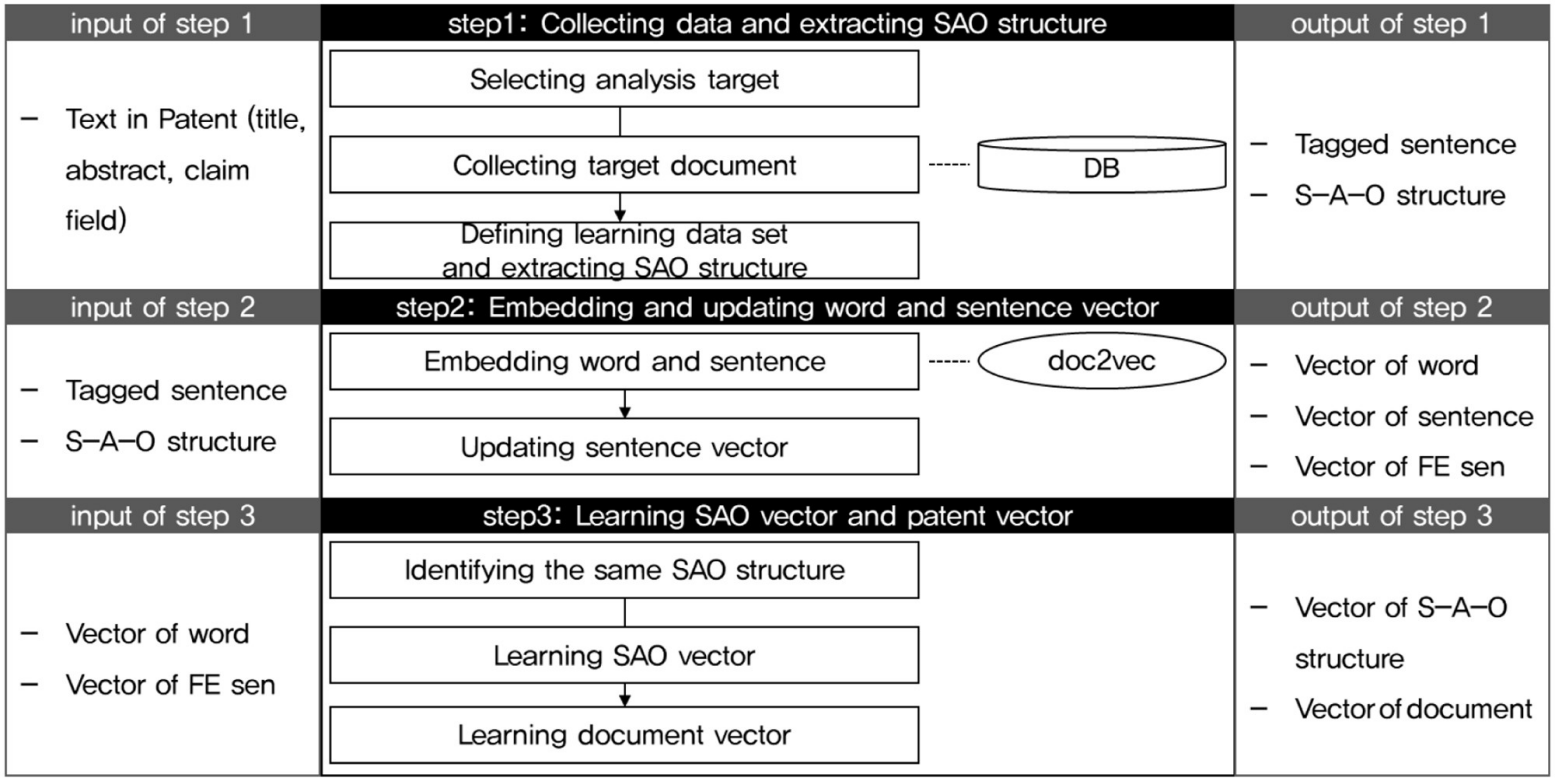
Research framework: The framework of this study has three steps. The first step is to collect data and extract the SAO structure, which will be used in future steps. And the second step is to extract words and sentence vectors using doc2vec algorithm and update the sentence vector. In the final step, SAO vectors and sentence vectors are obtained.

SAO2Vec consists of three phases, as shown in Fig 2. For each step of the framework, the input is shown on the left and the output on the right. First, technical documents are collected, and SAO structures are extracted from them based on part-of-speech tagging. In this step, the collected data and the SAO structures extracted by pos tagging are used to create the learning data for the next step. In this step, the input data is the text in the document and in case of patent documents, it can be the title, abstract and claims of a patent. In addition, the outputs of step 1, tagged sentences and SAO structures become the input data of step 2. In step 2, after deriving word and sentence vectors through Doc2Vec, the algorithm updates the sentence vectors using the word vectors and SAO structures. After obtaining the sentence vector and the word vector with Doc2Vec, the sentence vector is updated to increase the cosine similarity with the words in the SAO structure. The results of step 2 are vectors of word, sentence, and function-emphasized sentence(FE sen) vectors. The updated sentence vectors are FE sen vectors because the algorithm highlights and learns the meaning of the functions. Finally, using the vectors of word and FE sen, the algorithm learns the sentences that contain the SAO structures in order to obtain the SAO and document vectors. The learning process uses the basic idea of Word2Vec: If two words have high co-occurrence, they are semantically similar and have similar vectors. As a result of these steps, the vector of the SAO structure and the document can be obtained.

### 3.2 Collecting data and extract SAO structure

In Step 1, the data are selected and collected, the datasets for learning are derived from the collected data, and the SAO structures are extracted. First, the collected technical documents are preprocessed and separated into sentences. Then, the document number and sentence number are indexed so that each sentence is derived from a technical document and defined as a learning dataset to enable the word and sentence vectors to be learned.

Extracting SAO requires a parser, which analyzes a series of symbols based on regular grammar rules. Researchers in computer linguistics use parsers to construct parsing trees that show semantic relationships—either as a formal analysis of sentences or as a string of words in a component of a sentence. Many researchers use the Stanford Parser because it is available as an open-source program [49]. In this study, we used the Stanford Parser to separate sentences, then extracted the SAO structure through a series of linguistic algorithms. The index number of patent (patent_num), the index number of sentences (sentence_num), and the index number of SAOs (SAO_num) are then indexed on the extracted SAO structure.

**Fig 3:**
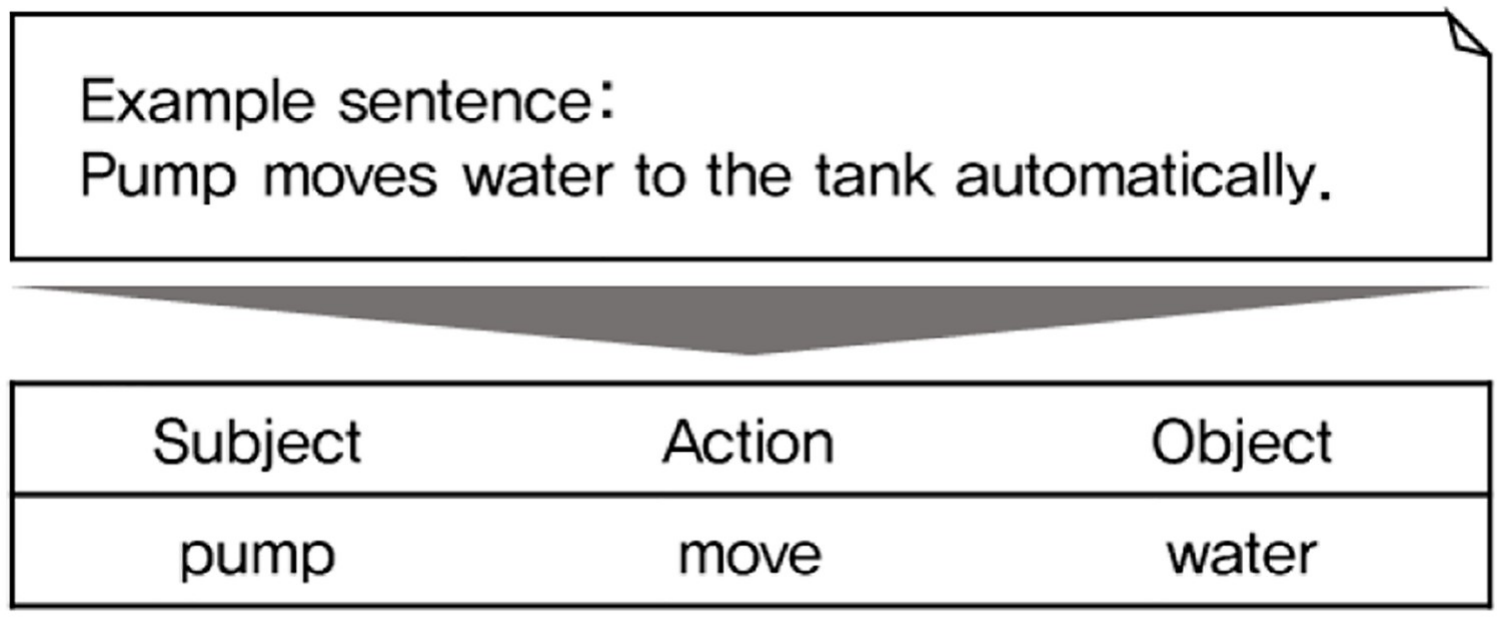
Example of SAO extraction.

Fig 3 shows an example of an extracted SAO structure. In the example sentence (“Pumps moves water to the tank automatically”), the thing that is doing the action (the “pump”) is the subject. The action is what the “pump” does (“moves”), and the object to be acted upon is “water.” The example sentence is as simple as possible because only one SAO structure can be extracted; however, several SAO structures can be extracted from a sentence, depending on its structure. In addition, structures that are omitted from one of the three elements can also be extracted.

### 3.3 Word and sentence vector embedding and updating

Step 2 produces a word vector and a sentence vector; the latter is updated using the previously obtained SAO structure. This process allows for the algorithm to obtain the FE sentence vector, which highlights the function of the technology. The concept of FE sentence vector proposed in this paper is a function-emphasized sentence vector, as mentioned earlier. It is so named because it focuses on reflecting the meaning of a function of technology that can be expressed as an SAO structure, rather than an existing sentence vector that focuses on the meaning of words and context in a sentence. The process of obtaining the FE sentence vector is shown below. First, to obtain word and document vectors that reflect the context, the Doc2Vec procedure (which applies the same weight to all words in the technical document) is conducted. The resulting sentence vector is then updated using the SAO structure from Step 1. Eq 1 is adapted to this framework; when a sentence is entered as input, the algorithm uses the probability that the output word will be the same as the observed SAO-structure word as an objective function. To maximize that function, the weight matrix of sentences must first be updated, as shown in Line 7 of Algorithm 1. This is done to place the sentence vector closer to the word vector from the SAO structure, thus better reflecting the meaning of the SAO structure. The learning process is the same as that of Doc2Vec. In the Doc2vec, the word vector and sentence weight vector is updated by using the target word as input data and the words in the window size as correct answers. In this study, the word and sentence vector is updated by using the target word as the input word and the word shown in the SAO structure as the correct answer data rather than the word within the window size.

**Table:** 

**Algorithm 1** Sentence vector update	
1:	For each document do
2:	For each sentence do
3:	If SAO exists then
4:	For each S, A, O do
5:	*x_k_* ← S, A, O
6:	w ← S, A, O
7:	${{w^{d}}_{kl}}^{(new)}\leftarrow {{w^{d}}_{kl}}^{(old)}-\eta ∙\frac{\partial E\prime }{\partial {w^{d}}_{kl}}$
8:	End For
9:	End If
10:	End For
11:	$\vec{FEsen}\leftarrow {\dot{W}}^{d}$
12:	End For

Eq (1) is an objective function in the Doc2Vec algorithm. The left term is the probability that, when the word w_I_ appears, the peripheral word w_c,j_ is actually the observed word w_o,c_. Updates are made to maximize this probability.

Algorithm 1 is pseudocode that describes the process of updating the sentence vectors that are obtained through the Doc2Vec method. The updating process for all the words in the SAO structure of each document (as shown in Eq 1) is shown in Line 7. The resulting vector is the FE sentence vector.

Because the SAO structure represents the function within the sentence [38], the FE sentence vector defines the updated sentence vector. Therefore, these sentence vectors better express the document’s technical content than do the vectors obtained from the Doc2Vec algorithm.

**Fig 4:**
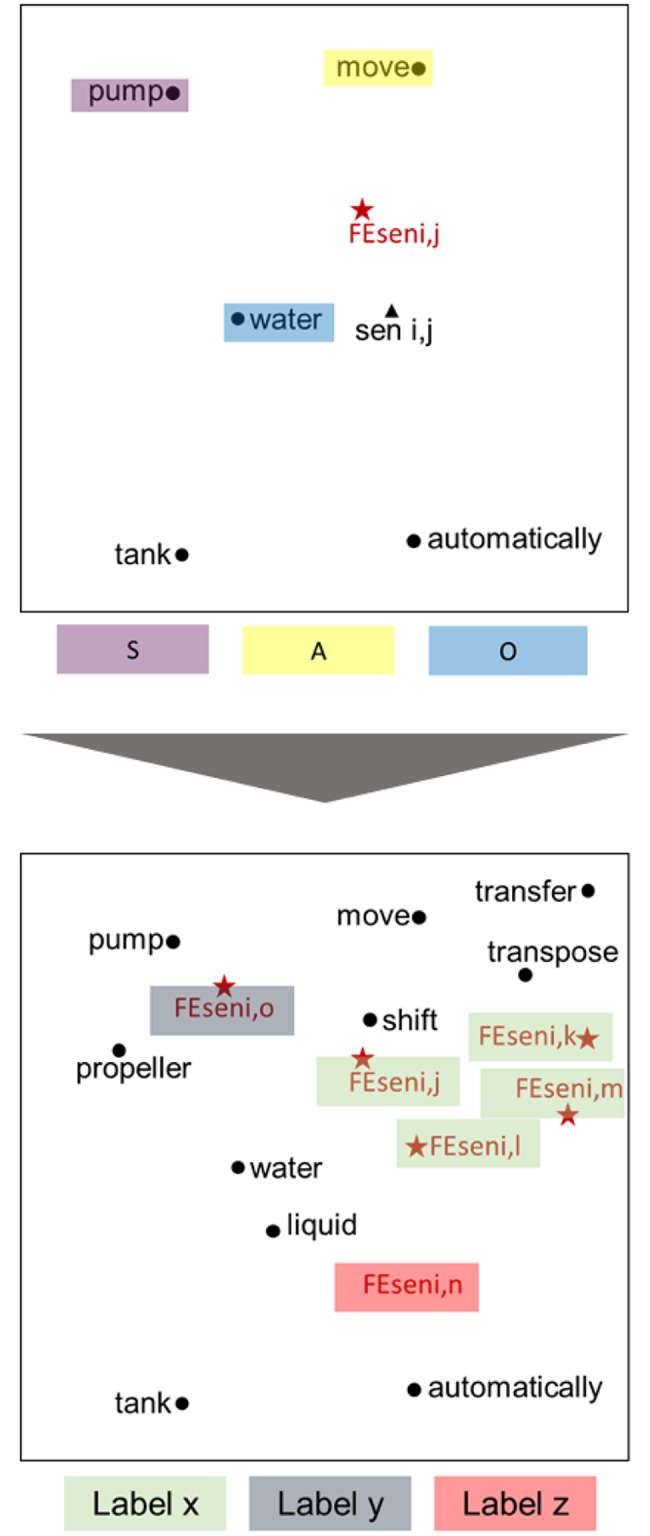
Word and sentence vector embedding and updating.

Fig 4 shows an example visualization of the results for an FE sentence vector created by updating the sentence vector. In the left panel of Fig 4, each word from the example sentence “Pump moves water to the tank automatically” is shown as a vector within a circle. The sentence vectors are in triangles. The FE sentence vector that is obtained through the update process is within a star; this is relatively close to the words “pump,” “water,” and “move”—which form the SAO structure for the original sentence vector.

### 3.4 Obtaining the SAO and document vectors

Step 3 is the calculation of the SAO and document vectors. To identify semantically identical SAO structures, the cosine similarity of each pair of SAOs is calculated, resulting in a similarity value. There are a number of ways to calculate vector similarity: Minkowski distance (e.g. Manhattan and Euclidian distance), Mahalanobis distance, cosine similarity, and so on. In Word2Vec (and Doc2Vec), the magnitude of the vector indicates the frequency of a word’s (or document’s) appearance, and the direction of the vector indicates the meaning of the word (or document) [50]. The similarity of meaning is reflected using cosine similarity, which measures the similarity of the vector directions. If the calculated similarity exceeds the cutoff value, then the two SAOs define the same SAO structure and have the same label; this helps to identify the sentences that contain the SAO list and the SAO.

Algorithm 2 is pseudocode that identifies and labels shared SAO structures. A 1:1 comparison of all the extracted SAO structures define the similarity matrices, and the SAO structures whose values exceed a set threshold are defined as identical. The cosine similarity equation described in Eq 2 involves determining a similar degree between vectors using the cosine values of the angles between those vectors in internal space. In this paper, we defined the similarity between two words (x and y) as the cosine similarity between those words’ vectors. In addition, we calculated the similarity of SAO vectors based on cosine similarity. First, for each component of SAO (as shown in Line 8), we calculated the cosine similarity for each pair of subjects, pair of actions, and pair of objects. Then, as shown in Line 9, we multiplied these three values to calculate the similarity between the two SAO structures. The cosine similarity for each pair of components has a value between 0 and 1, so the similarity of the resulting SAO structures also has a value between 0 and 1. We defined the threshold for similarity using trial and error. As a result, we obtained labels for SAO structures, as shown in Fig 4.

**Table:** 

**Algorithm 2** Identify same SAO structure	
1:	c ← 0
2:	For each SAO do
3:	If SAOx don’t have a label
4:	label_SAOx ← c
5:	For each SAO
6:	If SAOy don’t have a label
7:	calculate similarity(SAOx,SAOy)
8:	similarity(SAOx,SAOy) ← cosim(Sx,y)* cosim(Ax,y)* cosim(Ox,y)
9:	cosim(Kx,y) ← $\frac{(\overset{⃑}{W_{K\;\text{in}\;\text{SAOx}}}∙\overset{⃑}{W_{K\;\text{in}\;\text{SAOy}}})}{∥\overset{⃑}{W_{K\;\text{in}\;\text{SAOx}}}∥∙∥\overset{⃑}{W_{K\;\text{in}\;\text{SAOy}}}∥}$
10:	If similarity > t
11:	label_SAOy ← c
12:	End If
13:	End If
14:	End For
15:	c ← c+1
16:	End If
17:	End For

$$sim(x,y)=\frac{\langle x,y\rangle }{∥x∥∥y∥}$$

*where x* = (*x*_1_, *x*_2_, …, *x*_*n*_), *y* = (*y*_1_, *y*_2_, *…*, *y*_*n*_)

$$∥x∥=\sqrt{\sum_{i=1}^{n}{\left(x_{i}\right)}^{2}},\langle x,y\rangle =\sum_{i=1}^{n}x_{i}y_{i}$$ (2)

When learning an SAO vector, the value of the FE sentence vector for the sentence with the SAO should be used. This value contains not only the meanings of the SAO’s words but also the meanings of all of the words in the SAO’s sentence. Using this method, the SAO vector can be used to obtain vectors whose words or contexts would not be considered when using only SAO. A document’s SAO vectors are then calculated to distinguish among the meanings of the various SAO structures used within the document. In other words, the SAO vector is derived by averaging the vectors for all the sentences in which a particular SAO structure appears. The resulting SAO vector reflects the meanings of all the sentences in which the SAO is written in a document. This enables quantitative analysis of the SAO structure in various documents that have similar but not identical meanings. The calculation process is shown in Algorithm 3.

**Table:** 

**Algorithm 3** Calculate SAO vector	
1:	For each sentence do
2:	For each label do
3:	If sentence label = = label then
4:	add $\overset{⃑}{FEsen}$ to group ‘label’
5:	End If
6:	End For
7:	End For
8:	For each group ‘label’
9:	$\overset{⃑}{\text{SAO}}\leftarrow \text{average}(\overset{⃑}{FEsen}\left|\text{group}\;‘\text{label}’\right)$
10:	End For

**Fig 5:**
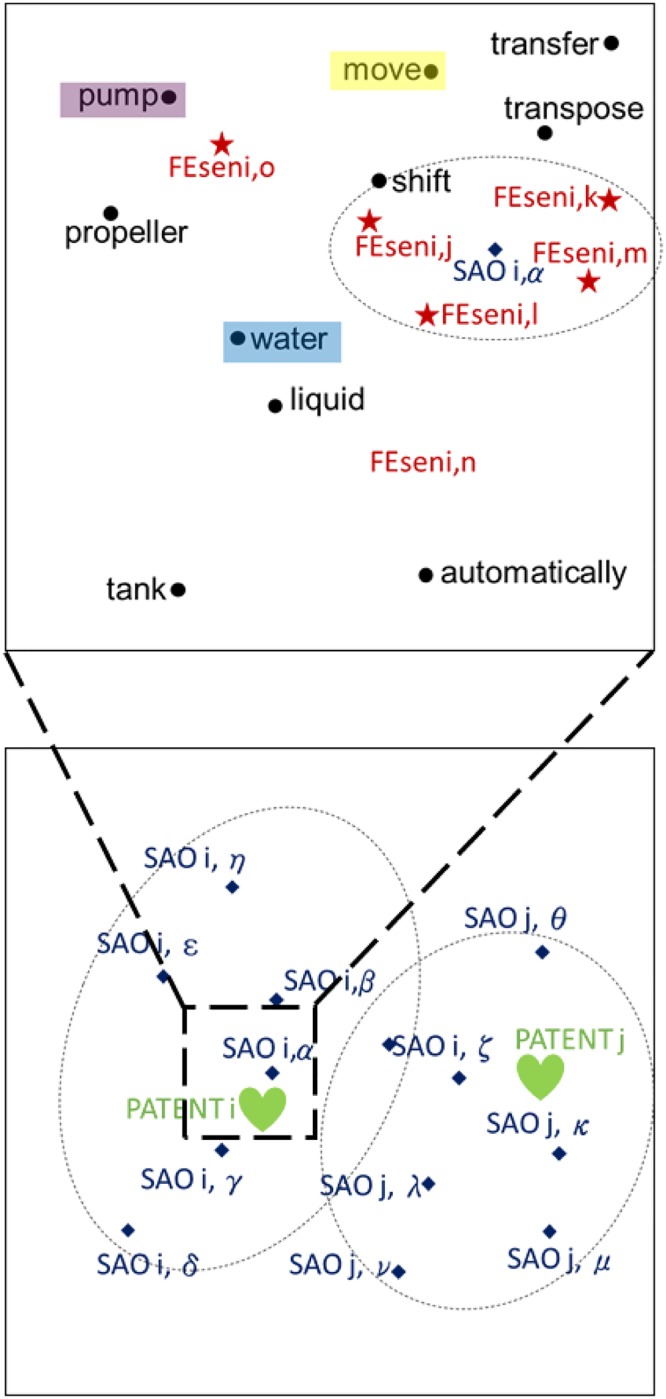
An algorithm for determining activeness of each document.

The document vector is calculated using the SAO vector, as shown in Algorithm 4, and the resulting document vector is located closer to the word vector of the document’s SAO structure than to the vector that the Doc2Vec method produces. The document vector’s emphasis is on technical features. Fig 5 is a visualization of these processes.

**Table:** 

**Algorithm 4** Calculate document vector	
1:	For each document do
2:	$\overset{⃑}{\text{DOC}}\leftarrow \text{average}(\overset{⃑}{FEsen}\left|\text{group}\;‘\text{document}’\right)$
3:	End For

Algorithms 3 and 4 are pseudocode examples that represent the process of obtaining the SAO and document vectors. The same label is obtained to define the mean value of the FE sentence vector and of the SAO structure vector; in the same process, the average vector value for all the SAO structures in the document is defined as the vector for that document. The obtained SAO and document vectors are the results of the SAO2Vec algorithm, which accounts for the technical features and contexts.

## 4. Experimental results

### 4.1 Datasets

To demonstrate the validity and applicability of the suggested algorithms, we conducted an analysis of patents in the IoT field. A patent is a technical document that provides good database availability, diverse coverage, and information; a patent also indicates the technology’s purpose and provides details about it [51]. Therefore, in this study, we conducted an analysis of patent documents. Because the abstract is considered the most important part of a patent, many previous scholars have only analyzed patents’ abstracts [52]. However, because abstracts do not include all the information about a technology’s purpose or the problem that it is meant to solve, we analyzed the full texts of the patents in this study [34, 53].

As mentioned in the Introduction (Section I), this study is an analysis of wireless IoT communication and network technologies. The IoT technology field shows a high growth rate and patent registration rate. In addition, IoT technology includes a wide range of applications that can be used as a foundation technology of many fourth industrial technologies such as healthcare and smart factories. Therefore, although one IoT technology field was selected as an analysis field, it is valid as verification data because it is a technology field that can represent various technology fields well. In addition, the electronic communication technology field, such as the IoT technology field, is a field in which a function of where and how a subject works is relatively clearly expressed, and thus, it is easy to extract SAO. We collected the patent documents from the United States Patent and Trademark Office (USPTO) patent database, as shown in Table 2.

**Table 2:** Collect data.

DB	US Patent
Keyword	IoT: device-to-device, D2D, machine-to-machine, M2M, internet and things, IoT Transmission and wireless communications networks: transmission, transfer, data, information, signal
Period	20080101 ~ 20180101
Results	941
USPTO link	http://patft.uspto.gov/netacgi/nph-Parser?Sect1=PTO2&Sect2=HITOFF&u=%2Fnetahtml%2FPTO%2Fsearch-adv.htm&r=0&p=1&f=S&l=50&Query=ttl%2F%28IoT+OR+%22device-to-device%22+OR+D2D+OR+%22device+to+device%22+OR+%22machine-to-machine%22+OR+M2M+OR+%22machine+to+machine%22+OR+%28internet+AND+things%29+OR+%28Transmission+AND+wireless+AND+communication+AND+network%29%29+and%0D%0Aisd%2F1%2F1%2F2008-%3E1%2F1%2F2018.&d=PTXT

### 4.2 Extracting SAO structure

We derived the learning dataset by preprocessing the name, abstract, and all claims in patent documents related to wireless communication and network technologies in the IoT field. A learning dataset is a sentence unit in which the patent_num and sentence_num are indexed, and the preprocessing steps involve changing capitalized letters to lowercase ones and then removing stop words. Examples of learning datasets are shown in Table 3.

**Table 3:** Example of prepared learning data in Iot field.

Patent num^a^	Sentence num^b^	Sentence
2	102	transmit power level control for the discovery signal transmission
4	218	the non-transitory computer-readable storage device of claim, wherein the instructions further cause the use to perform operations to: encode the discovery signal for the additional transmissions using additional discovery resources from at least a second sub-discovery resource pool of the plurality of sub-discovery resource pools

^a^Patent num is the index number of a patent.

^b^Sentence num is the index number of the sentence from a patent.

We used the Stanford Parser to extract the SAO structures from the learning dataset. These structures are closely related to the parsing algorithm that distinguishes the subject, verb, and object within a sentence. We extracted 79,303 SAO structures; an example SAO structure from the sentences is shown in Table 4. An extracted SAO structure is a function of the technology that the patent describes. As an example, the SAO structure from Sentence 218 of the fourth patent in Table 3 is one of the claims related to that patent. This SAO structure describes a stable, computer-readable storage device. The algorithm extracted three SAOs from this sentence: the 475th, 476th, and 477th SAO structures. The meaning of the 475th SAO structure is that the device in the patent will perform the operation. The meaning of the 476th SAO structure is that the device’s operation will encode the discovery signal. Finally, the meaning of the 477th SAO structure relates to a transaction using additional discovery resources. These SAO structures summarize the patent’s claim well. The SAO structure may also contain three elements of the SAO, along with an omitted element; we used all four structures in this study. In addition, depending on the number of phrases in a sentence, it could have several structures; in this study, we extracted and used all SAO structures from all syntax depths equally.

**Table 4:** Example of SAO structure extracted by the learning data.

Patent num^a^	Sentence num^b^	SAO num^c^	S^d^	A^e^	O^f^
2	218	475	instructions	perform	operation
		476	operation	encode	discovery signal
		477	additional transmission	use	additional discovery resource

^a^Patent num is the index number of a patent.

^b^Sentence num is the index number of sentence from a patent.

^c^SAO num is the index number of SAO structure from a patent.

^d^S is the subject word in the SAO structure.

^e^A is the action word in the SAO structure.

^f^O is the object word in the SAO structure.

### 4.3 Word and sentence vector embedding and updating

**Fig 6:**
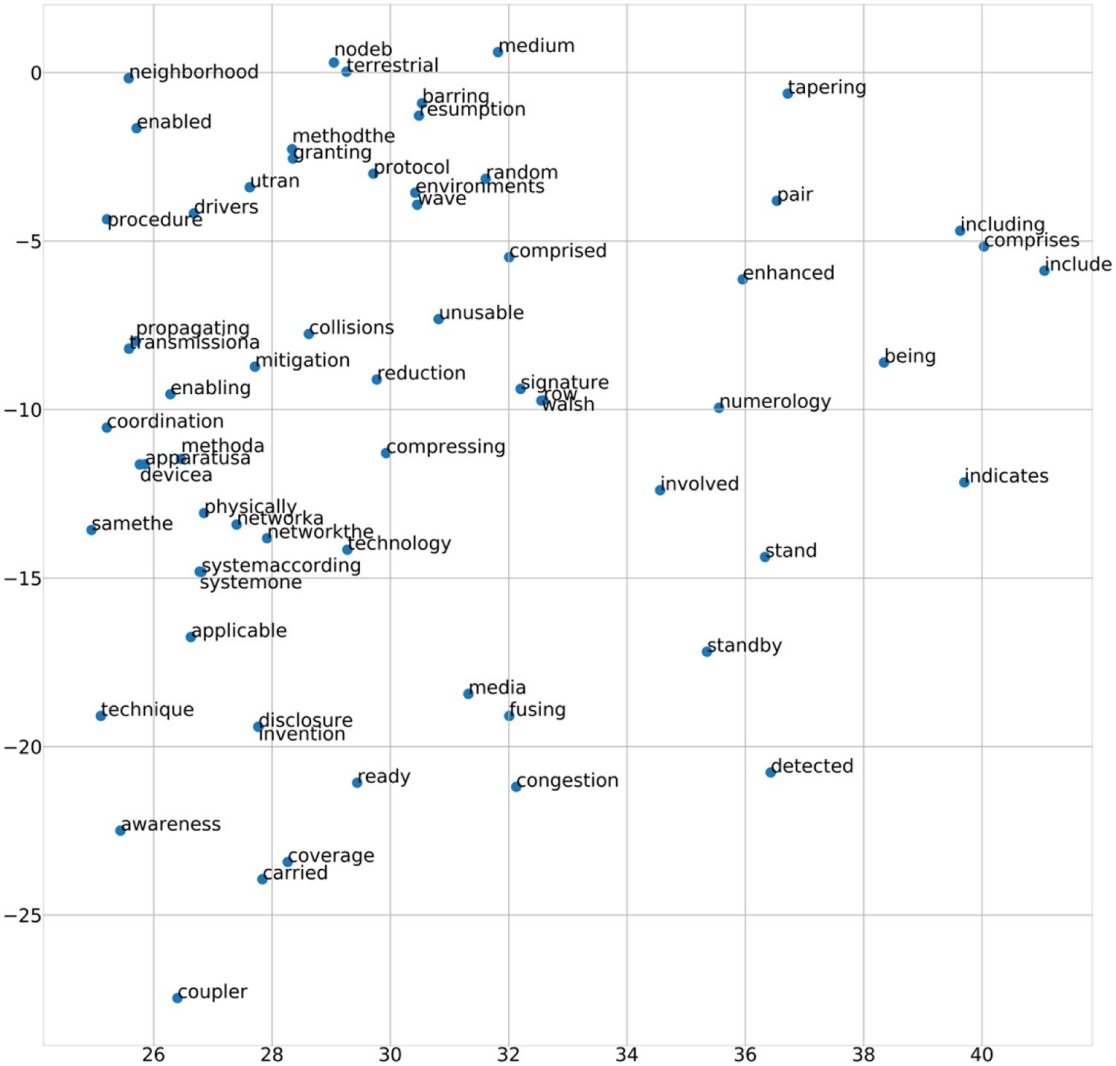
Visualization of word vector ‘technique’.

The first step prior to obtaining the vector of the SAO structure is to obtain the vectors of the words and sentences in the full document’s dictionary. Using the dm model of the Doc2Vec Package, we obtained the vector values of the words and sentences. We set the window size—which defines how the algorithm looks at co-occurrence when obtaining vector values—to 8. We calculated the vector using only 200 dimensions and required five or more appearances in the documents. These parameters are the result of choosing using trial and error to find the most descriptive parameters. The resulting word and sentence vectors from Doc2Vec are transferred to the same dimension, and a two-dimensional visualization of them using the t-distributed stochastic neighbor embedding (TSNE) algorithm is shown in Fig 6.

Table 5 lists the words with high cosine sensitivity from the example words and Doc2Vec. The word “technique” is followed by “fusing,” “technology,” “converging,” “procedures,” and “discovery.” The fusing technique involves a combination of data derived from various sensors or sources, each of which has less uncertainty than the existing method, in which each data source is used individually. This is one of the most important IoT technologies for transmitting and using data from various sensors. “Technology” is also a synonym for “technique,” and the word “disclose” describes the difference between the current technology and the corresponding patent. In addition, the words “resource,” “subframe,” and “wireless” all appeared with the words “transmission” or “transmitting.” Thus, Doc2Vec produced the proper word and sentence vectors. Fig 6 shows an example visualization of the word “technique” and its surrounding words. If a word has low cosine intensity around it, then it can not be exactly visualized in two dimensions because the angles of the cosine similarity are equal. However, the presence of “fusing,” “converging,” and “disclosure” near the word “technique” indicates that the results are valid.

**Table 5:** Top 5 words similar with “technique” and “transmission” using Doc2vec result.

word	Cosine similarity^a^				
	top1	top2	top3	top4	top5
Technique	fusing	Technology	converging	Procedure	disclosure
transmission	communication	transmitting	resource	subframe	wireless

^a^Cosine similarity top x means that the cosine similarity with the given word is x^th^ most similar.

Next, we performed an update to highlight the meaning of the SAO structure in the sentence vector and thus to calculate the FE sentence vector. This requires a sentence vector, an SAO structure derived from that sentence, and a word vector for the words comprising that SAO structure. Table 6 shows the average similarity value of the sentence vectors prior to the 102nd sentence, as well as the words in the updated SAO structure. These values indicate that the mean similarity of the FE sentence vector is greater than that of the sentence vector. This means that the FE sentence vector is located closer to the vector of the subject, action, and object words than to the sentence vector, which in turn means that the FE sentence vector better reflects the function’s SAO structure than the ordinary sentence vector does.

**Table 6:** Example of the average similarity between sentence vector and word vector.

Avg_similarity^a^	S vector^b^	A vector^c^	O vector^d^
Sen vector	- 0.03385	0.00255	- 0.01029
FE sen vector	0.02575	0.00946	0.04026

^a^Avg_similarity is the average of similarity in all pairs of vectors

^b^S vector is the average of similarity in all pairs of Subject vectors

^c^A vector is the average of similarity in all pairs of Action vectors

^d^O vector is the average of similarity in all pairs of Object vectors

**Fig 7:**
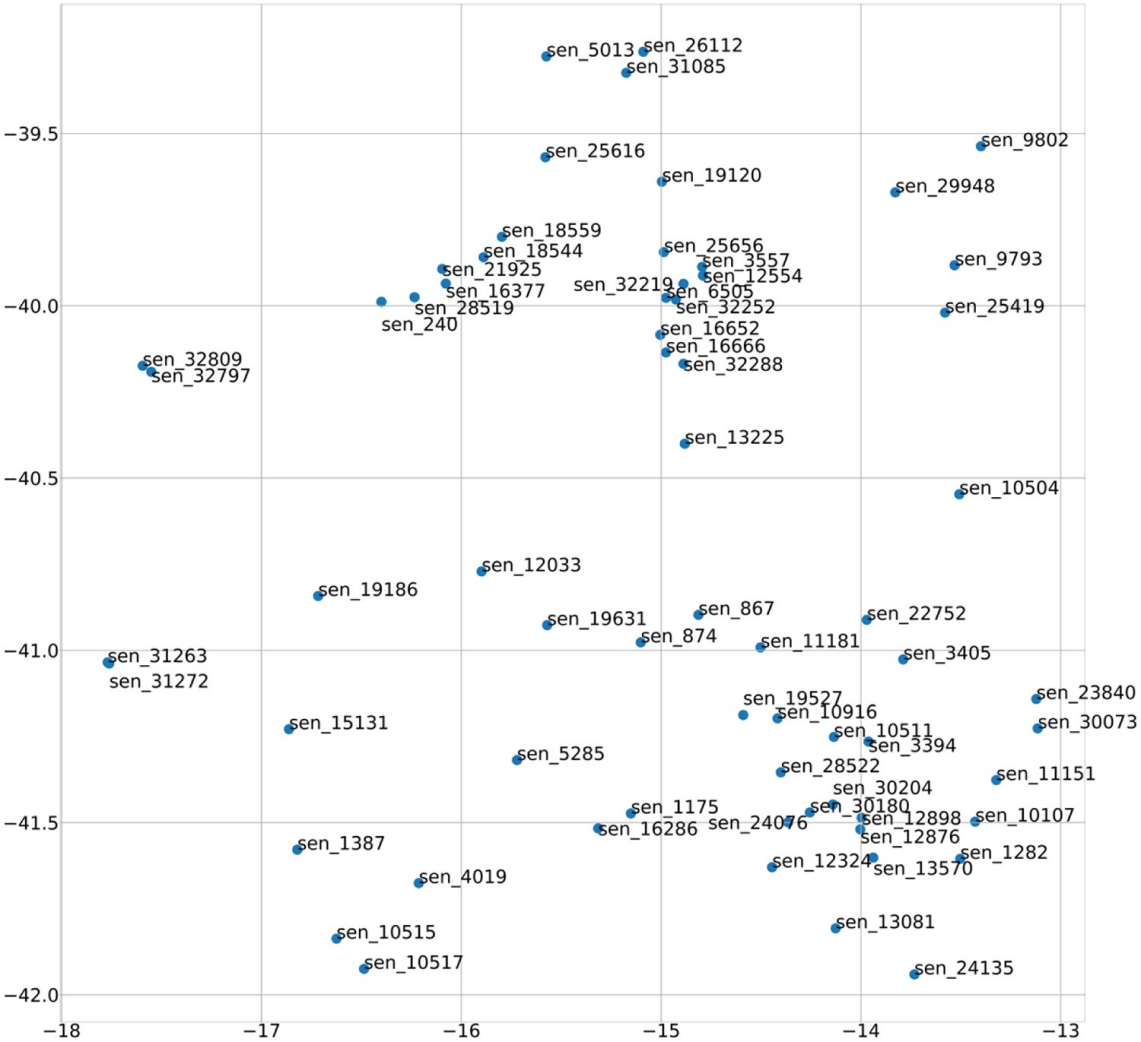
Visualization of the 5285th sentence vector.

**Fig 8:**
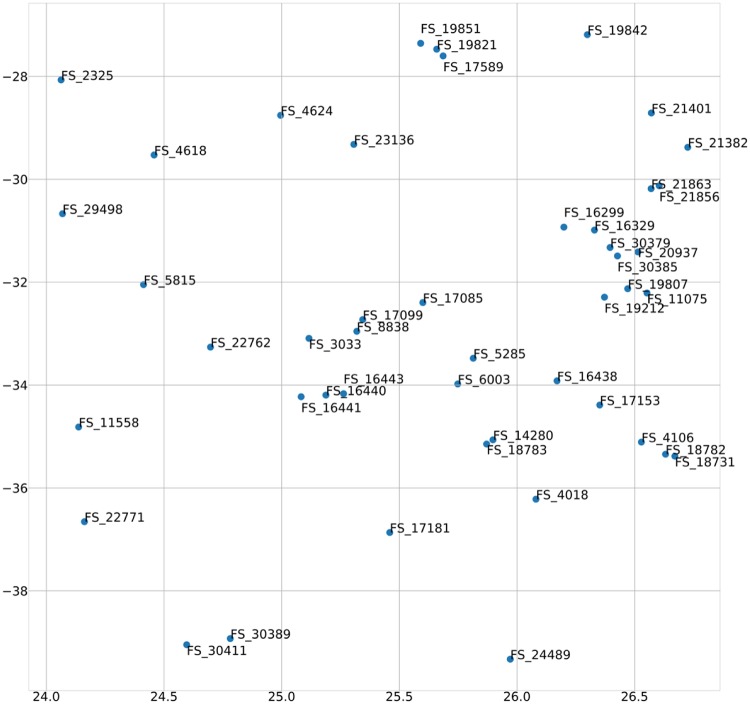
Visualization of the 5285th FE sentence vector.

As an example, we can compare the sentence vector with the FE sentence vector. Sentence 5285 describes the transmitter: “A method for facilitating device to device (D2D) discovery, the method comprising: transmitting, by an evolved node B (eNB), a device to device (D2D) discovery configuration to one or more user equipment (UEs), the D2D discovery configuration associated with a set of periodically occurring time-domain periods,” As shown in Fig 7, the sentence whose vector is most similar to that of the fifth sentence is Sentence 16,286: “a controller configured to process data that is to be transmitted to the server, according to the received information about the transmission authority, the communicator transmits the data to the server.” On the other hand, as shown in Fig 8, the sentence whose FE sentence vector is most similar to that of Sentence 5285 is Sentence 6003: “The user device then allocates a transmission resource a D2D discovery signal within the discovery time interval according to the d2d discovery configuration.” Sentence 6003, which describes the transport resources at the time of the data transmission, is technologically more similar to Sentence 5285 than to Sentence 16,286, which describes the controller. As such, the FE sentence vector has more technical meaning than a vector obtained through the traditional Doc2Vec method.

### 4.4 Learning SAO vector and patent vector

Prior to learning the SAO vector using the previously obtained FE sentence vector, the algorithm labeled 79,303 SAO structures. Using the vectors of the previously obtained words, it calculated the cosine similarity of the SAO structure and labeled it. SAO structures with the same label are structures with similar meanings. The algorithm thus generated 5901 labels; Table 7 shows that the same SAO structure appeared in various patents and sentences.

**Table 7:** Example of the labeled SAO structure: Label 32, 93.

Label^a^	Patent num^b^	Sentence num^c^	SAO num^d^	S^e^	A^f^	O^g^
32	2	218	475	instructions	perform	operation
	2	230	496	instructions	perform	task
	405	14671	34778	-	perform	operation
93	2	218	476	operation	encode	Discovery signal
	4	206	444	operation	encode	information
	267	9736	22707	operation	comprise	signal

A total of 5901 labels were derived from 79,303 SAO structures. Label 32 is a set of 106 SAO structures, and label 93 is a set of 149 SAO structures, with each of the three examples represented in the table.

^a^Label is index of the unique SAO structure.

^b^Patent num is the index number of a patent.

^c^Sentence num is the index number of sentence from a patent.

^d^SAO num is the index number of SAO structure from a patent.

^e^S is the subject word in the SAO structure.

^f^A is the action word in the SAO structure.

^g^O is the object word in the SAO structure.

Then, using the FE sentence vector of the labeled SAO structure, the algorithm created a dataset to calculate the SAO vector. Because the vector values according to SAO structures should be calculated for one document, the vector value of SAO Structure 32 in Document 2 is calculated using the FE sentence vector of the n sentences that have that SAO structure. The resulting SAO vector is shown in Table 8.

**Table 8:** Example of SAO vector: Sentence 1134, 2261, 206 in patent 218.

Patent_num^a^	Sentence_num^b^	SAO vector^c^ (dim = 200)
218	1134	(0.491999, 1.288587, 0.317469, …, -0.432819, 0.162475, 0.000578)
218	2261	(0.530694, 0.459372, 0.197969, …, -1.144108,-0.387236,-0.423902)
…	…	…
218	206	(0.634885, 1.22471, 0.558733, …, -0.544525,-0.155806,0.17755)

Patent No. 218 contains sentences with 35 SAO structures, three of which are represented in the table.

^a^Patent num is the index number of a patent.

^b^Sentence num is the index number of sentence from a patent.

^c^SAO vetor is the 200 dimensional vector of the SAO structure

The SAO vector derived using SAO2Vec reflects the meaning of the words other than the SAO, which differs from the analysis based on the existing SAO approaches. The SAO vector contains more information on the documents and contexts than do the existing SAO structures, so it can better represent the meaning of the functions—including the key findings related to the technology—than can a sentence vector that is calculated with the traditional Doc2Vec method. In addition, as shown in Table 6, the FE sentence vector is more similar than the ordinary sentence vector to the words in the SAO structure. In addition, analyses that use existing SAO structures are limited in that the same SAO structure is treated in the same way, even if it appears in different documents. However, in this study, the SAO vector was calculated for each document. In other words, the same SAO structure can be distinguished across documents based on the presence of different meanings or contexts.

Table 9 shows the vectors of the SAO Structure 32, which are derived from various patent documents. They have different vector values even though they use the same SAO structure. This method thus can be particularly useful when analyzing patent documents, as all such documents basically describe techniques for solving problems, so the SAO structures of all patent documents have unique meanings or contexts, even when they employ the same structures. The SAO vector thus helps analysts to perform analyses that reflect this distinction.

**Table 9:** Uses of an SAO vector with the same label.

Patent_num^a^	Sentence_num^b^	SAO vector^c^ (dim = 200)
294	32	(0.597186, 0.546221, 0.097256, -0.112597, 0.121704, 0.521426, …)
303	32	(0.595341, 0.474635, 0.180812, 0.745539, 0.436642, 0.22292, …)
520	32	(0.58665, 0.508009, -0.08149, 0.07969, 0.273633, 0.452418, …)

Label 32 appeared in a total of 73 patents, and the table shows three examples and the SAO vector of Label 32 in that document.

^a^Patent num is the index number of a patent.

^b^Label is index of the unique SAO structure.

^c^SAO vetor is the 200 dimensional vector of the SAO structure

We calculated the document and SAO vectors in the same way. The vectors that we used to calculate the document vector are also the SAO vectors in the corresponding document. An example of a resulting document vector is shown in Table 10.

**Table 10:** Example of document vector: Patent 1, 2, 3.

Patent_num^a^	Document vector^b^ (dim = 200)
1	(0.166322247,0.4541985,0.055633331, …, -0.023517468,0.156254383,-0.128338305)
2	(0.051742255, 0.193958759, 0.32022656, …, -0.05796744, 0.433337986, 0.312557369)
941	(0.302176, 0.811281897, 0.201013195, …, -0.176340402, -0.392004782, -0.195887195)

Document vectors were calculated for a total of 941 documents, and the table shows three examples.

^a^Patent num is the index number of a patent.

^b^Document vector is the 200 dimensional vector of the document.

## 5. Discussion and implications

The results of the SAO2Vec algorithm are an SAO vector and a document vector. In this section, we examine how the resulting vectors work in practice.

### 5.1 Validation

The proposed SAO2Vec aims to derive a vector that reflects the technological information of a document well. Thus, the document vector, which is made from the SAO2Vec algorithm, can be considered for investigating technical elements. Therefore, in the case of technical documents, the SAO2Vec will produce more accurate results than other various algorithms in generating document vectors. To verify that the results of SAO2Vec reflect the technical contents better than existing models, we compared the results of clustering tasks against other approaches such as Doc2Vec and SAO frequency vectors. The spectral clustering algorithm was used as a model of the clustering task, and the input was a vector of documents obtained from SAO2Vec, DOC2Vec, and SAO frequency algorithms. As the output, a cluster of patent documents is shown, and the accuracy of each algorithm is to be remarked with the correct answer data of the representative IPC code of the cluster. In order to use the spectral clustering algorithm, the first step is to derive a similarity matrix of documents by using the input vector of each document. The document similarities are calculated from the document vectors of the SAO2Vec, Doc2Vec and the SAO structure frequency. The method of calculating the similarity was used for each algorithm. The document vectors from Doc2Vec and SAO2Vec both used cosine similarity, but the document vectors from SAO frequency used Euclidean-distance similarity. This is because the Doc2Vec and SOA2Vec algorithms learned the word vectors by increasing the internal value between word vectors, so the resulting vectors were best explained using cosine similarity. Document vectors based on SAO frequency use only the frequency of SAO structures, and these vectors are proportional to the number of SAO structures, with features referring to SAOs. Therefore, we decided that Euclidean-distance similarity was the most appropriate technique.

The resulting matrices are shown in Tables 11, 12 and 13. Each similarity value is between 0 and 1; values close to 0 mean that the two patents are dissimilar, and values close to 1 mean that the two patents are similar. Thus, in a matrix, the (x, x) coordinates—comparing parent x to itself—thus equate to the largest similarity value (1), as this is the similarity of a patent with itself. For example, in Tables 11, 12 and 13, the (x, x) value has the highest similarity value (1) because it is comparing the document to itself. In addition, Tables 11, 12 and 13 have distinct values (0.030, 0.048, and 0.054, respectively) for (1, 4)—the similarity of Document 1 with Document 4. This indicates that each method produced a distinct value for the similarity between Document 1 and Document 4. Thus, the accuracy of each approach can be calculated using the similarity matrices in Tables 11, 12 and 13.

**Table 11:** Example of document similarity matrix using SAO frequency.

similarity	doc1	doc2	doc3	doc4	doc5
doc1	1	0.027	0.032	0.030	0.033
doc2	-	1	0.034	0.033	0.036
doc3	-	-	1	0.041	0.048
doc4	-	-	-	1	0.043
doc5	-	-	-	-	1

**Table 12:** Example of document similarity matrix using Doc2Vec.

similarity	doc1	doc2	doc3	doc4	doc5
doc1	1	0.106	0.089	0.048	0.178
doc2	-	1	0.148	0.576	0.184
doc3	-	-	1	0.424	0.304
doc4	-	-	-	1	0.307
doc5	-	-	-	-	1

**Table 13:** Example of document similarity matrix using SAO2Vec.

similarity	doc1	doc2	doc3	doc4	doc5
doc1	1	0.367	0.056	0.054	0.127
doc2	-	1	0.199	0.684	0.579
doc3	-	-	1	0.460	0.354
doc4	-	-	-	1	0.429
doc5	-	-	-	-	1

As mentioned earlier, patent data have the label like patent codes and we used criteria based on the international patent classification (IPC) codes of the patent documents to validate the document embedding results. The IPC code is the label that the patent applicants and the judges select the most suitable technology field for the patent. To show that the proposed embedding reflects technical information, this paper compares the clustering results using the embedding vector with the IPC codes. The model’s accuracy can explain how well it could form a cluster that represented IPC codes. We used the spectral clustering algorithm, which is a graph-based technique that uses undirected, weighted graphs. This algorithm creates clusters by increasing weights (in case that there are high similarities between two datasets) and lowering the weights (in case that the similarity is low). The benefit of this method is the consistency of results, which is not obtained in the parametric model-based approach. Spectral clustering requires a pre-determined number of groups, k, to be applied. In this study, the number of k for the results obtained by each methodology was calculated using the eigengap heuristic methodology [54]. The eigengap heuristic methodology is based on perturbation theory and spectral graph theory to calculate the optimal number of clusters. The number of clusters was selected as a k value, which maximizes the eigengap, a difference between consecutive eigenvalues. Thus, the values of k became k = 53 in the SAO2Vec approach, k = 76 in the Doc2Vec method, and k = 34 in the SAO frequency approach. After clustering the patent documents using this algorithm with the driven k, we checked to determine whether the patent documents included in each cluster were similar. Although the IPC code consists of sections, classes, subclasses, groups, and subgroups, classes and subclasses are considered in this validation section. One patent could have multiple IPC codes, using all IPC codes that each patent had. Five IPC codes with a high frequency of IPC codes in the obtained clusters were defined as the cluster’s representative technology IPC codes. If the IPC of a patent does not belong to the representative IPC of the cluster to which the patent belongs, the embedding result of the patent document was interpreted as a failure to adequately represent the patent document. Table 14 shows the results of the spectral clustering using vectors for the patents acquired with each method. Of the total 914 documents, vectors derived from SAO2Vec correctly classified 875 documents, vectors from Doc2Vec classified 849 documents and finally, vectors from SAO Frequency correctly classified 407 documents. Based on this result, SAO2Vec was 3.1% better than Doc2Vec and 115.0% better than SAO frequency.

**Table 14:** Patent clustering using spectral clustering.

	SAO2vec	Doc2vec	SAO frequency
Accuracy	875/941 = 0.93	849/941 = 0.90	407/9441 = 0.43

The results of the patent clustering show that the vector based on SAO frequency performed the worst and that the vector based on Doc2Vec performed the best.

### 5.2 Implications

Analyzing technical documents using the SAO vector and the patent vector (which is obtained as a result of the SAO2Vec algorithm) enables quantitatively digitized analysis and simultaneous consideration of the technology’s function and context. The context-based analysis involves using all sentence structures other than the SAO structure (which existing SAO structures ignore), meaning that the information present in the technical documentation is used more often. In particular, the SAO vector that results from SAO2Vec can be used to distinguish between SAO structures that are identical but that are used in different patent documents (and thus with different meanings). Although existing SAO analyses do not take this into account, the results of SAO2Vec show that it is possible for analysis to reflect this. This method has the advantage of being able to greatly reduce the passive, professional-oriented analysis of the existing SAO structure, which can save a lot of resources and time. Compared to the Doc2Vec algorithm, this method produces more accurate vectors for technical documents, resulting in higher accuracy for the clustering and other analyses. The results in Section 5.2 show that the parent vector from SAO2Vec has higher accuracy than the parent vector from Doc2Vec. This is because the existing Doc2vec used the co-occurrence of only words in the same window. Although the Doc2vec can express the general meaning of words well expressed, the problem-solving method of technology cannot be sufficiently presented.

However, SAO2Vec has several limitations: First, the threshold is that the accuracy depends on the process used to extract the SAO structures, which is the most important aspect of learning in SAO2Vec. The SAO structure is not simply a structure that comprises a verb or an object (as in the structure of a sentence); it is an architecture that represents the function of technology and that indicates how the subject takes action against the object. Therefore, distinguishing between sentence structures limits the accuracy of SAO2Vec, depending on the algorithm that is used to determine the SAO structure, the parser accuracy, and the differentiated sentence structure. In addition, the learning rate must be manually selected in the process of updating the sentence vector with the FE sentence vector. The algorithm alone can not obtain the most appropriate learning rate; the user instead must select the learning rate that produces the most descriptive results, based on trial and error. Because SAO2Vec’s produces the extracted and digitized information from the text rather than a single answer, it is also limited in that the results—even though they may be useful—can not be quantitatively verified. Finally, it also has the limitation of presenting inconclusive results because we used only a single dataset in this study.

## 6. Conclusions

In this study, we developed SAO2Vec, an algorithm for embedding SAO structures based on the Doc2Vec learning method. The algorithm starts by collecting technical documents and extracting their SAO structures. Then, the word and sentence vectors are extracted through Doc2Vec; in an update process, the sentence vector that emphasizes the meaning of the technology is then derived. The SAO vector is obtained by calculating the FE sentence vector, and the document vector is obtained by calculating the SAO vector. As a result of analyzing patents in the IoT field, we were able to group and calculate the similarity in the meaning of technical elements and contexts. This process took less effort and less time than the existing analytical method. In addition, the results of the proposed approach were 3.1% more accurate than Doc2Vec and 115.0% more accurate than the SAO frequency method, as shown in Table 14.

However, as mentioned above, this study has some limitations, including problems with the NLP accuracy, the learning rate in vector-learning processes, and text mining. To address these points, scholars could conduct further studies on this subject so as to address the aforementioned limitations and increase accuracy. First, by improving NLP, the algorithms that extract SAO structures can be improved. In this study, we used the Stanford Parser because it is open source and the simplest algorithm for extracting SAOs. Using more advanced parsers and more sophisticated algorithms could improve the quality of the SAOs and, in turn, increase accuracy. Second, this experimental study is meant to determine the most descriptive learning rate. In the update process for changing learning rates or making the sentence vectors into FE sentence vectors, the resulting vectors’ quality can be experimentally verified when using a learning rate. In addition, to compensate for the limitation of being difficult to verify, a study of SAO2Vec’s applications can be actively conducted so as to demonstrate its usefulness. In this paper, we selected the IoT field for illustration because it is the most representative area where the proposed framework can be applied. First, the IoT field has been regarded as a promising area that can lead the fourth industrial revolution. Thus, there are incredible needs for the extensive investigation of patents in the field. Second, the data where the proposed approach, SAO2vec works well should have a descriptive structure from which the SAO structure is successfully drawn and the collected patents of IoT field generally provide a clear description of the technology. Thus, technology fields that can clarify the functions of tools or components are proper to the application of the proposed approach. For example, mechanical technology and information technology will be appropriate fields for the SAO2vec. However, since biological technology and chemical technology normally process technology or based on complex theory, the proposed approach can hardly show high performance. Thus, scholars in further studies will be able to analyze multiple datasets in order to determine whether this framework can work in various contexts and situations. Additional data that can be analyzed can be categorized into formats and technical fields. The format of the data can include patents, papers, technical reports, and the like. Although patents and technical reports generally consist of many SAOs, academic papers mainly have a detailed description of theoretical logic and experiment. Thus, the proposed process can be successfully applied to patents and technical reports rather than academic papers. In addition, the technical fields for the application of this approach may include artificial intelligence or display fields in addition to the IoT field applied in this study. In particular, mechanical technology will be a potential technology field that the SAO2vec can be applied to because we can clearly derive the functions from equipment or devices. In this regard, the direction of research can be divided into several points. For example, there is a need to conduct a practical study comparing a technical data set (such as patents and technological reports) with a data set of non-technical documents (such as social data). In this study, we just applied the proposed approach to patents among technical data, but in the case of technical reports, a better SAO structure can be obtained because the sentences are shorter than patents. In addition, further research can build a model that can be applied to other fields having very different technical characteristics such as chemistry, physics, and computer science. Currently, the framework works well only in the fields of telecommunications and machinery where the SAO structure is well derived, but further research will be able to compensate for the limited application of the technique. In addition, since this study is based on the Doc2vec model, there is a limitation that the homonym is not considered well. The Doc2Vec model is a pre-training learning model, which is contrary to end-to-end learning, which aims to process the desired task, a trend of recent representation learning, in one model. Therefore, the research that applies the concept of SAO structure to a representation model other than Doc2Vec can also be conducted.
